# Reduction of a Prolapsus Uteri after Sixteen Years’ Continuance

**Published:** 1842-03

**Authors:** 


*Reduction of a Prolapsus Uteri after sixteen years' continuance.*—M. DURANT records an interesting case of this in the Transactions of the Medical Society of Ghent. The womb protruding beyond the external parts, and covered by the inverted vagina, presented a globular tumor, round, and contracted at its origin into the form of a circular appendix. The *os uteri* was clear at its inferior part. The tumor at its middle part was fifteen and a half inches in circumference. Its external surface was brownish red, and covered with crusts and ulcerations. The long continuance of the affection had seriously injured the general health of the patient—she was pale and emaciated, and subject to sleeplessness, and cramps of the stomach.

Mr. Durant, before attempting reduction, kept the patient on light diet, and at rest in bed in a proper position; at the same time dressing the tumor with opiated emollient fomentations. Its surface speedily softened, and the crusts fell off, leaving superficial sores. After six days of this treatment the operation was performed. It having been ascertained that the rectum and bladder were empty, the patient was placed in the position most advantageous for the entrance of the womb. M. D. then introduced the right forefinger into the os uteri, and burying it, pushed upwards in the axis of the tumor, which itself was placed in the axis of the true pelvis—then retaining the uterus in its place with the left hand, withdrew the finger, and, repeating this manipulation with gentleness, just as one turns the finger of a glove outside in, accomplished the reduction in less than half an hour. He then inserted into the vagina a sponge, cut into the form of a cylinder, and saturated with an emollient decoction, the thick end being highest up, and a cord attached to the other, for the purpose of removing it at pleasure. This sponge-pessary was retained in its place by means of compresses, and the T bandage. The patient did well, speedily gaining flesh and strength. During the after-treatment, which continued for about six weeks, emollient and astringent lotions were employed, and an ordinary-sized caoutchouc ring-shaped pessary was used, the saturated sponge and the injections being passed through its centre.—*Am. Jour. Med. Sci.*, from *Journ. de Med. et de Chirurg. Prat.* Feb. 1841.

